# Assembly and analysis of the complete mitochondrial and chloroplast genomes of *Vigna reflexo-pilosa*

**DOI:** 10.1371/journal.pone.0325243

**Published:** 2025-06-11

**Authors:** Panthita Ruang-areerate, Suparat Pinsupa, Wasitthee Kongkachana, Thippawan Yoocha, Phakamas Phetchawang, Peeraphat Paenpong, Prakit Somta, Kularb Laosatit, Sithichoke Tangphatsornruang, Wirulda Pootakham

**Affiliations:** 1 National Center for Genetic Engineering and Biotechnology (BIOTEC), National Science and Technology Development Agency (NSTDA), Pathum Thani, Thailand; 2 Department of Agronomy, Faculty of Agriculture at Kamphaeng Saen, Kasetsart University, Kamphaeng Saen Campus, Nakhon Pathom, Thailand; Jeju National University, KOREA, REPUBLIC OF

## Abstract

*Vigna reflexo-pilosa* can be found in both wild and cultivated forms. It is the only tetraploid species in the genus *Vigna* in Fabaceae, occurring through hybridization between *Vigna hirtella* and *Vigna trinervia*, with the chromosome number of 2n = 4x = 44. *V. reflexo-pilosa* provides an invaluable gene pool for improving cultivated *Vigna* crop varieties. This study aimed to report the complete mitochondrial and chloroplast genomes of *V. reflexo-pilosa*. A total of 6,496,297 raw reads were generated from *V. reflexo-pilosa* using the long-read PacBio technology. The complete mitochondrial genome was assembled into a linear structure with a total length of 370,913 base pairs (bp) with 45.20% GC content. It contains 32 protein-coding genes, 18 transfer RNA genes, and 3 ribosomal RNA genes. A total of 520 RNA editing sites were detected in 30 protein-coding genes. The *V. reflexo-pilosa* mitochondrial genome shared large colinear blocks with *Vigna radiata* as compared to nine other mitochondrial genomes in Fabaceae. In addition, *Vigna hirtella* (male parent) and *Vigna trinervia* (female parent) were sequenced using the MGI sequencing technology. The complete chloroplast genome of *V. reflexo-pilosa, V. hirtella*, and *V. trinervia* was assembled into a circular structure with a total length of 150,967, 151,915 and 151,226 bp, respectively. All three chloroplast genomes consist of 128 genes. We found no evidence of shared genes between the mitochondrial and chloroplast genomes of *V. reflexo-pilosa*. Comparison of the three *Vigna* chloroplast genomes showed high levels of similarity between *V. reflexo-pilosa* and *V. trinervia*, revealing maternal inheritance of the chloroplast genomes. Based on both mitochondrial and chloroplast genes, phylogenetic trees showed that *V. reflexo-pilosa* is closely related to *V. radiata*. These genomes enhance our understanding of mitochondrial and chloroplast evolution of *V. reflexo-pilosa* and are valuable genetic resources in legumes.

## Introduction

The genus *Vigna* belonging to the family Fabaceae includes over 100 species of five subgenera, which are *Ceratotropis*, *Haydonia*, *Lasiosporon*, *Plectotropis* and *Vigna* [1,2]. Among them, *Vigna* species in the subgenus *Ceratotropis* distribute across Asia, thus they are known as Asian *Vigna* [3]. Seven Asian *Vigna* species including *Vigna aconitifolia* (moth bean)*, Vigna angularis* (azuki bean)*, Vigna mungo* (black gram)*, Vigna radiata* (mung bean), *Vigna reflexo-pilosa* (créole bean), *Vigna stipulacea* (Minni Payaru) and *Vigna umbellata* (rice bean) have been domesticated owing to their rapid growth and adaptability to various cropping systems [3]. *V. reflexo-pilosa*, which is a wild legume and a neglected crop distributed in Asia, is originated from interspecific hybridization from two genome donors, *Vigna hirtella* and *Vigna trinervia* [4,5]. It is a tetraploid legume species with the chromosome number of 2n = 4x = 44, whereas other *Vigna* species are diploid legume species with the chromosome number of 2n = 2x = 22 [3–5].

Mitochondrion plays a crucial role in energy production in animal and plant cells. In general, mitochondrial genomes are circular double-stranded DNA molecules that are various in contents, sizes and structures, especially in plants [6,7]. The mitochondrial genomes of legumes in Fabaceae are approximately 400 kb in length [8–12]. For example, the mitochondrial genomes of cultivated mung bean (*V. radiata*) and wild mung bean (*V. radiata* var. sublobata) are 401 and 403 kb in length, respectively [8,9]. The mitochondrial genomes of common bean (*Phaseolus vulgaris*) and wild soybean (*Glycine soja*) are also 390 kb and 400 kb in length, respectively [10,11].

Chloroplast is an organelle within plant cells that is specifically used for photosynthesis. Chloroplast genomes commonly consist of a large single-copy region (LSC), inverted repeats (IRs) and a single small-copy region (SSC) [12–22]. In legumes, chloroplast genomes are approximately 150 − 155 kb in length and encode about 126 − 130 genes [12–19]. For instance, the chloroplast genome of *V. radiata* and *Glycine max* is approximately 151 and 152 kb in length and consist of 127 and 130 genes, respectively [15,17].

Next-generation sequencing (NGS) is used to study genomes and genes associated with interesting traits. In plants, NGS has been successfully utilized to sequence complete mitochondrial genomes [9–12,23,24] and chloroplast genomes [12–22].

In this study, we sequenced, assembled, and annotated the mitochondrial genome of *V. reflexo-pilosa* and the chloroplast genomes of *V. reflexo-pilosa, V. hirtella*, and *V. trinervia* using the PacBio and MGI sequencing technologies, respectively. We analyzed mitochondrial RNA editing sites, repeats and SSRs, and compared our mitochondrial genome with related legume species. Transfer sequences between mitochondrial and chloroplast genomes of *V. reflexo-pilosa* were also evaluated. Moreover, we conducted a phylogenetic analysis of *V. reflexo-pilosa* using mitochondrial and chloroplast genes.

## Materials and methods

### Plant materials, DNA extraction, and sequencing

We used DNA of the créole bean (*V. reflexo-pilosa* var. *reflexo-pilosa*) accession AusTRCF30263 that was previously used in Pootakham et al. [5]. According to the previous study, DNA was extracted using CTAB buffer [25] and 25:24:1 phenol, chloroform, and isoamyl alcohol. DNA was precipitated using absolute ethanol and washed with 70% ethanol. DNA was resuspended in 10 mM Tris-HCl (pH 8.0) after air drying. DNA was purified using the Ampure PB beads kit (Pacific Biosciences, Menlo Park, CA, USA), and the integrity of DNA was assessed using the Pippin Pulse Electrophoresis system (Sage Science, Beverly, MA, USA).

To sequence mitochondrial *(V. reflexo-pilosa)* and chloroplast genomes (*V. reflexo-pilosa, V. hirtella*, and *V. trinervia*), DNA libraries and sequencing were prepared following the previous studies [5,26]. Briefly, the SMRTbell Express Template Prep Kit 2.0 was used to create the PacBio SMRTbell library from the high molecular weight DNA template, and the PacBio Sequel system (Pacific BioSciences, Menlo Park, CA, USA) was used to sequence the libraries and to produce raw reads for assembling a mitochondrial genome. Whole-genome libraries for the chloroplast genome sequencing were constructed following the MGIEasy Universal DNA Library Prep Set Instruction Manual (MGI Tech, San Jose, CA, USA) and sequenced on the DNBSEQ-G400 using the MGISEQ-2000RS Sequencing Flow Cell v3.0 following the manufacturer’s protocol.

### Assembly and annotation

The long-read PacBio raw data were assembled using Canu v1.9 with the default parameters [27]. To extract mitochondrial contigs, all contigs were blasted against three published mitochondrial *Vigna* genomes, *V. radiata* (HM367685), *V. angularis* (NC 021092), and *V. unguiculata* (MW448464), using BLASTN [28]. Several mitochondrial contigs were identified and chosen when they have the longest length and cover all mitochondrial genomes. Moreover, the selected mitochondrial contig was blasted against known mitochondrial genes of *V. radiata* using BLASTN. Finally, genes and ribosomal RNAs (rRNAs) were identified using the mitofy software v2012 [29], and transfer RNAs (tRNAs) were detected using tRNAscan-SE v1.23 with the default parameters [30].

Chloroplast genomes were assembled and annotated using the GetOrganelle software v1.7.7.1 [31] and the web application GeSeq [32], respectively. Chloroplast protein-coding genes and rRNAs were identified using ARAGORN v1.2.36 [33] in the GeSeq software. Chloroplast tRNAs were identified using tRNAscan-SE v1.23. Furthermore, OGDRAW v1.3.1 [34] was used to display the circular structure of the chloroplast genomes.

To investigate chloroplast-derived regions, the mitochondrial genome of *V. reflexo-pilosa* was blasted against its chloroplast genome using BLASN, with a threshold of 1e-10 and >90% identity.

### Identification of RNA editing sites, repeats, and SSRs

To identify candidate RNA editing sites, we used transcriptome data from a previous study [5]. The transcriptome data was mapped to the *V. reflexo-pilosa* mitochondrial genome using minimap v2.17 [35]. The RNA-seq mapping result was visualized using IGV v2.8.10 [36], and then RNA editing sites were manually checked for changing C-to-T (the plus stand of a gene) or G-to-A (the minus stand of a gene) with changes by at least 10%.

Repeat sequences in the *V. reflexo-pilosa* mitochondria genome were identified using the web application REPuter [37]. In addition, the simple sequence repeats (SSRs) of the *V. reflexo-pilosa* mitochondrial genome were detected using MISA v2.1 [38] with the default parameters (mononucleotide (the number of repeats = 10), dinucleotide (6), trinucleotide (5), tetranucleotide (5), pentanucleotide (5), and hexanucleotide (5)).

### Comparative analysis of mitochondrial genomes

Structure variations in mitochondria genomes between *V. reflexo-pilosa* and three other *Vigna* species (*V. angularis*, *V. radiata*, and *V. unguiculata*) as well as seven plant species in Fabaceae (*Glycine max*, *Glycine soja*, *Medicago polymorpha*, *Phaseolus vulgaris*, *Robinia pseudoacacia*, *Senna occidentalis*, and *Trifolium grandiflorum*) were preformed using MAUVE v20150226 [39] and the D-GENIES web application [40]. The mitochondrial genomes of the ten species are available in the NCBI database with accession numbers (see in the method of phylogenetic analysis)

Furthermore, the sequence divergence of shared fragment mitochondrial sequences and 14 conserved genes between *V. reflexo-pilosa* and three other *Vigna* species (*V. angularis*, *V. radiata*, and *V. unguiculata*) was estimated using dnaSP v.6.12.03 [41].

### Comparative analysis of chloroplast genomes

Sequence divergence among three chloroplast genomes (*V. reflexo-pilosa*, *V. trinervia*, and *V. hirtella*) was investigated using mVISTA with the Shuffe-LAGAN mode [42]. The *V. reflexo-pilosa* chloroplast genome was used as a reference.

### Phylogenetic analysis

Phylogenetic analyses with the maximum likelihood (ML) method were carried out using MEGA X [43] based on both mitochondrial and chloroplast genes. The best-fit model was a GTR + I + G model that was evaluated in the mitochondrial and chloroplast gene sets using the best DNA/protein model tool in MEGA X. Node supports were performed with 1000 bootstrap replicates. *Ginkgo biloba* is used as an outgroup species.

For mitochondrial phylogeny, we used 14 mitochondrial genes including *atp9, ccmB, ccmC, cob, cox1, cox3, nad3, nad4, nad6, nad7, nad9, rps3, rps4* and *rps12*, which were shared in *V. reflexo-pilosa* and 28 plant species. The 28 mitochondrial genomes from the NCBI database are 10 legumes in Fabaceae (*Glycine max*: NC_020455, *Glycine soja*: NC_039768, *Medicago polymorpha*: MW971562, *Phaseolus vulgaris*: NC_045135, *Robinia pseudoacacia*: MW448465, *Senna occidentalis*: NC_038221*, Trifolium grandiflorum*: NC_048501, *Vigna angularis*: NC_021092, *Vigna radiata*: NC_015121, and *Vigna unguiculata*: MW448464) and 18 other plants (*Arabidopsis thaliana*: NC_037304, *Capsicum annuum*: NC_024624, *Citrullus lanatus*: NC_014043, *Cucumis sativus*: NC_016005, *Cucurbita pepo*: NC_014050, *Eucalyptus grandis*: NC_040010, *G. biloba*: NC_027976, *Gossypium barbadense*: NC_028254, *Luffa acutangula*: NC_050067, *Nicotiana tabacum*: NC_006581, *Oryza sativa*: NC_066488, *Populus alba*: NC_041085, *Salix purpurea*: NC_029693, *Solanum lycopersicum*: NC_035963, *Sorghum bicolor*: NC_008360, *Zea luxurians*: NC_008333, *Zea mays*: NC_007982, and *Zea perennis*: NC_008331).

For chloroplast phylogeny, we used 54 chloroplast genes, including *atpA*, *atpB*, *atpE*, *atpF*, *atpH*, *atpI*, *ccsA*, *clpP*, *matK*, *ndhB*, *ndhC*, *ndhE*, *ndhF*, *ndhG*, *ndhH*, *ndhI*, *ndhJ*, *petA*, *petB*, *petD*, *petG*, *petL*, *petN*, *psaA*, *psaB*, *psaC*, *psaI*, *psaJ*, *psbA*, *psbC*, *psbD*, *psbE*, *psbF*, *psbH*, *psbI*, *psbJ*, *psbK*, *psbM*, *psbN*, *psbT*, *psbZ*, *rbcL*, *rpl20*, *rpl23*, *rpl2*, *rpl36*, *rpoA*, *rpoB*, *rps2*, *rps3*, *rps4*, *rps7*, *rps8*, and *ycf3*, were shared in the chloroplast genome of three *Vigna* species from our study (*V. reflexo-pilosa, V. hirtella*, and *V. trinervia*) and the 28 plant species, which are the same species in the construction of the mitochondrial evolutionary tree. The 28 chloroplast genomes from the NCBI database are 10 legumes in Fabaceae (*G. max*: PP712901, *G. soja*: NC_022868, *M. polymorpha*: NC_042848, *P. vulgaris*: NC_009259, *R. pseudoacacia*: NC_026684, *S. occidentalis*: OR478159, *T. grandiflorum*: NC_024034, *V. angularis*: NC_021091, *V. radiata*: NC_013843, and *V. unguiculata*: NC_018051) and the 18 other plants (*A. thaliana*: NC_000932, *C. annuum*: NC_018552, *C. lanatus*: NC_032008, *C. pepo*: NC_038229, *C. sativus*: NC_007144, *E. grandis*: NC_014570, *G. biloba*: NC_016986, *G. barbadense*: NC_008641, *L. acutangula*: MT381996, *N. tabacum*: NC_001879, *O. sativa*: NC_031333, *P. alba*: NC_008235, *S. purpurea*: NC_026722, *S. lycopersicum*: NC_007898, *S. bicolor*: NC_008602, *Z. luxurians*: NC_030301, *Z. mays*: NC_001666, and *Z. perennis*: NC_030300).

## Results

### Assembly and annotation of the *V. reflexo-pilosa* mitochondrial genome

**Fig 1:**
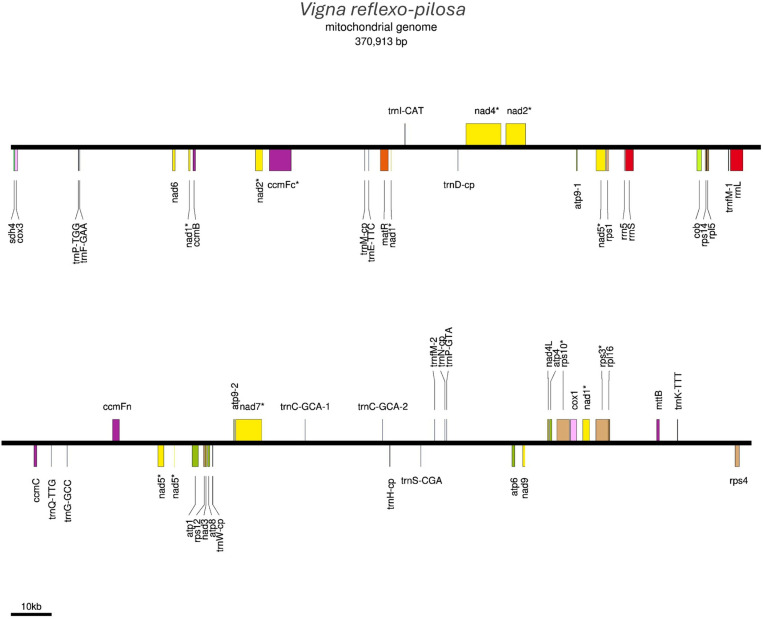
The linear structure of *V. reflexo-pilosa* mitochondrial genome. Protein-coding genes, tRNA genes, and rRNA genes are shown on the line.

A total of 6,496,297 PacBio long-reads were generated from *V. reflexo-pilosa* accession AusTRCF30263 and assembled into a mitochondrial genome. The complete mitochondrial genome of *V. reflexo-pilosa* was a linear structure and 370,913 bp in length (Fig 1). Four nucleotides, adenine (A), thymine (T), cytosine (C), and guanine (G), are present in percentages of approximately 27.52%, 27.28%, 22.62%, and 22.58%, respectively. The overall GC content is 45.20% (Table 1). The mitochondrial genome consisted of 53 genes, containing 32 protein-coding genes, 18 transfer RNA genes, and 3 ribosomal RNA genes as indicated in Fig 1 and Table 1.

**Table 1:** Genomic features of the *V. reflexo-pilosa* mitochondrial genome compared to other *Vigna* species.

Description	*V. reflexo-pilosa*	*V. radiata*	*V. angularis*
Total genome size (bp)	370,913	401,262	404,466
Protein-coding genes	32	32	32
rRNAs genes	3	3	3
tRNAs genes	18	16	16
GC percentage (%)	45.20	45.11	45.19

A total of 32 protein-coding genes were identified in the mitochondrial genome of *V. reflexo-pilosa* (Table 2). They were classified into nine groups including genes encoding ATP synthase, cytochrome c biogenesis, cytochrome c oxidase, cytochrome c reductase, maturase, NADH dehydrogenase, transport membrane protein, large ribosomal subunit, and small ribosomal subunit. Eight genes out of the 32 genes (*ccmFc, nad1, nad2, nad4, nad5, nad7, rps3,* and *rps10*) have introns as shown in Table 2. The sizes of protein-coding genes in the mitochondrial genome of *V. reflexo-pilosa* varied from 303 bp (*nad4L* and *rps14*) to 2002 bp (*nad5*).

**Table 2:** List of mitochondrial genes of *V. reflexo-pilosa.*

Group of genes	Genes
ATP synthase	*atp1, atp4, atp6, atp8, atp9* (x2)
Cytochrome c biogenesis	ccmB, ccmC, ccmFc*, ccmFn
Cytochrome c reductase	*cob*
Cytochrome c oxidase	*cox1, cox3*
Maturase	*matR*
Transport membrane protein	*mttB*
NADH dehydrogenase	nad1*, nad2*, nad3, nad4*, nad4L, nad5*, nad6, nad7*, nad9
Small ribosomal subunit (SSU)	rps1, rps3*, rps4, rps10*, rps12, rps14
Large ribosomal subunit (LSU)	*rpl5, rpl16*
ribosomal RNA (rRNA)	*rrnS, rrn5, rrnL*
transfer RNA (tRNA)	*trnP-UGG, trnF-GAA, trnM-CAU, trnE-UUC, trnI-CAT, trnD-GUC, trnW-CCA, trnfM-CAU* (x2)*, trnQ-UUG,* *trnG-GCC, trnC-GCA* (x2)*, trnH-GUG, trnS-CGA, trnN-GUU, trnP-GUA, trnK-UUU*

* The asterisk beside genes denotes intron-containing genes.

### Chloroplast genome assembly and annotation of *V. reflexo-pilosa, V. hirtella* and *V. trinervia*

**Fig 2:**
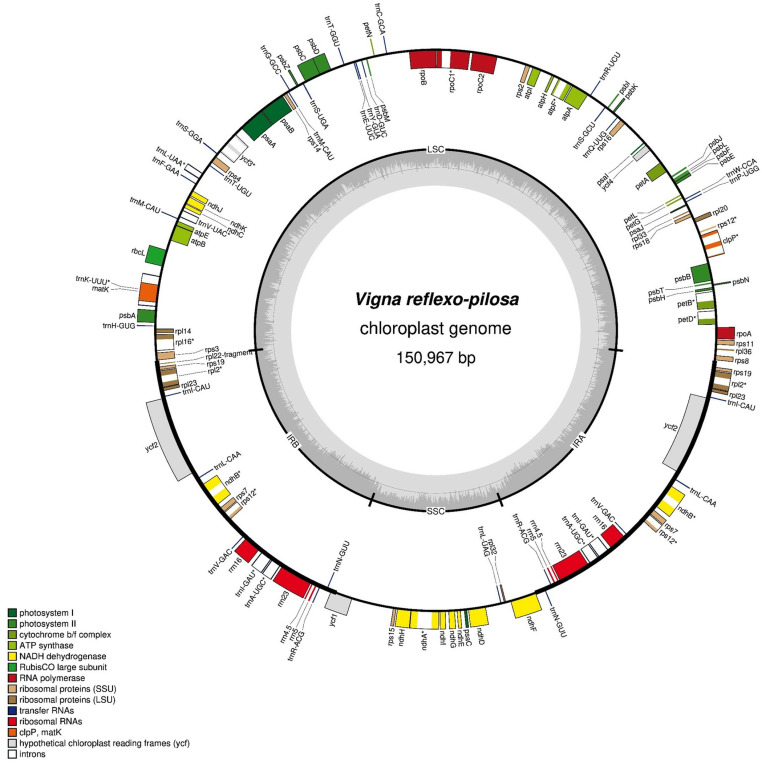
Circular structure of the *Vigna reflexo-pilosa* chloroplast genome. Known protein-coding genes, tRNA genes and rRNA genes are shown on the outside and inside of the circle. Colored genes were based on their functional groups.

The sizes of the complete chloroplast genomes of *V. reflexo-pilosa, V. trinervia,* and *V. hirtella* were 150,967 bp (Fig 2), 151,226 bp and 151,915 bp (S1 Fig), respectively. All three chloroplast genomes consist of 83 protein-coding genes, 36 tRNA genes, 8 rRNA genes, and a pseudogene. The chloroplast genome of *V. reflexo-pilosa* included a large single-copy (LSC) region of 80,704 bp, a small single-copy (SSC) region of 17,402 bp and two inverted repeat (IR) regions of 26,430 bp as shown in Table 3. The chloroplast genome of *V. trinervia and V. hirtella* also included the LSC region of 80,904 and 81,518 bp, the SSC region of 17,424 and 17,471 bp and the IR regions of 26,449 and 26,463 bp, respectively.

**Table 3:** Genomic features of *V. reflexo-pilosa, V. trinervia, and V. hirtella* chloroplast genomes.

Genome characteristics	*V. reflexo-pilosa*	*V. trinervia*	*V. hirtella*
Genome size (bp)	150,967	151,226	151,915
Large single copy (bp)	80,704	80,904	81,518
Inverted repeat (bp)	26,430	26,449	26,463
Small single copy (bp)	17,402	17,424	17,471
Genome GC content (%)	35.28	35.22	35.17
Protein coding genes	83	83	83
Transfer RNA genes (tRNA)	36	36	36
Ribosomal RNA genes (rRNA)	8	8	8
Pseudogene	1	1	1

A total of 128 protein-coding genes were identified in three *Vigna* chloroplast genomes and categorized into 17 functional groups (Table 4). These groups included genes encoding photosystem I subunit, photosystem II subunit, NADH dehydrogenase, cytochrome b/f complex subunit, ATP synthase subunit, rubisco, maturase, protease, envelope membrane protein, acetyl-CoA carboxylase, c-type cytochrome synthesis, large ribosomal subunit, small ribosomal subunit, RNA polymerase, ribosomal RNAs, transfer RNAs, and unknown function. Twelve genes out of the 128 genes have introns (*atpF, clpP1, ndhA, ndhB, petB, petD, rpl2, rpl16, rpoC1, rps7, rps12,* and *ycf3*).

**Table 4:** List of genes in the *V. reflexo-pilosa* chloroplast genome.

Category	Group of genes	Genes
Photosynthesis	Subunits of Photosystem I	*psaA, psaB, psaC, psaI, psaJ*
	Subunits of Photosystem II	*psbA, psbB, psbC, psbD, psbE, psbF, psbH, psbI, psbJ, psbK, psbL, psbM, psbN, psbT, psbZ*
	Subunits of NADH dehydrogenase	ndhA*, ndhB* (x2)*, ndhC, ndhD, ndhE, ndhF, ndhG, ndhH, ndhI, ndhJ, ndhK*
	Subunits of Cytochrome b/f complex	petA, petB*, petD*, petG, petL, petN
	Subunits of ATP synthase	atpA, atpB, atpE, atpF*, atpH, atpI
	Large subunit of rubisco	*rbcL*
Self-replication	Proteins of large ribosomal subunit	rpl2* (x2), rpl14, rpl16*, rpl18, rpl20, rpl22 (x2)*, rpl23* (x2)*, rpl32, rpl33, rpl36*
	Proteins of small ribosomal subunit	rps2, rps3, rps4, rps7* (x2), rps8, rps11, rps12* (x2), rps14, rps15, rps16**, rps19 (x2)
	RNA polymerase	rpoA, rpoB, rpoC1*, rpoC2
	Ribosomal RNAs	*rrn4.5, rrn5, rrn16, rrn23*
	Transfer RNAs	*trnA-UGC* (x2)*, trnC-GCA, trnD-GUC, trnE-UUC, trnF-GAA, trnG-GCC, trnH-GUG, trnI-CAU* (x2)*, trnI-GAU* (x2)*, trnK-UUU, trnL-UAG*, *trnL-CAA* (x2)*, trnL-UAA, trnM-CAU* (x2)*, trnN-GUU* (x2)*, trnP-UGG, trnQ-UUG, trnR-ACG* (x2)*, trnR-UCU, trnS-UGA, trnS-GGA, trnS-GCU, trnT-UGU, trnT-GGU, trnV-GAC* (x2)*, trnV-UAC, trnW-CCA, trnY-GUA*
Biosynthesis	Maturase	*matK*
	Protease	clpP1*
	Envelope membrane protein	*cemA*
	Acetyl-CoA carboxylase	*accD*
	C-type cytochrome synthesis gene	*ccsA*
Genes of unknown function	Conserved hypothetical chloroplast reading frames	*ycf1, ycf2* (x2), ycf3*, ycf4

Gene*, gene**, and gene (x2) denote one intron containing genes, two introns containing genes and a copy number of genes, respectively.

### RNA editing in the *V. reflexo-pilosa* mitochondrial genome

**Fig 3:**
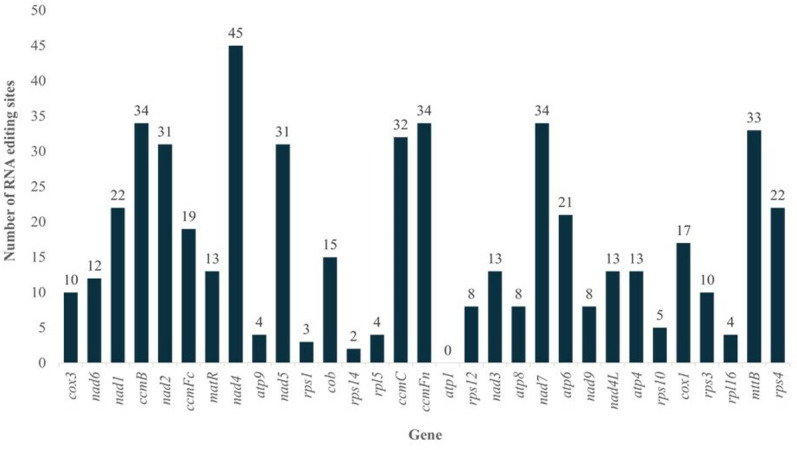
Distribution of RNA editing sites in 30 mitochondrial genes of *V. reflexo-pilosa.*

A total of 520 edited sites were identified in 30 protein-coding genes of the *V. reflexo-pilosa* mitochondrial genome (*atp4, atp6, atp8, atp9*, *ccmB, ccmC, ccmFc, ccmFn, cob, cox1, cox3, mttB, matR, nad1*, *nad2, nad3, nad4, nad4L, nad5, nad6, nad7, nad9, rpl5, rpl16, rps1, rps3, rps4*, *rps10, rps12,* and *rps14)*, containing edited C to T (G to A) base changes (Fig 3). However, no edited site was found in the *atp1* gene. The *nad4* gene contained the highest number, with 45 editing sites followed by *ccmFn* and *nad7* genes, each with 34 editing sites. Most genes typically start with the codon ATG. However, four genes (*mttB, nad1, nad4L,* and *rps10*) were edited at the second site of the start codon (ACG to ATG). Additionally, the editing site of *ccmFc* occurred at the first position of its stop codon (CGA to TGA).

### Repeats and SSRs in the *V. reflexo-pilosa* mitochondrial genome

A total of 50 repeats, consisting of 27 forward and 23 palindromic repeats, were detected in the mitochondrial genome of *V. reflexo-pilosa* (S1 Table). The repeats ranged from 40 to 357 bp in length.

A total of 37 SSRs were identified (S2 Table). Most of the mononucleotide repeats (96.70%) are composed of A/T. Among the monomeric SSRs, thymine (T) and adenine (A) repeats were the most abundant, accounting for 57.58% and 39.39% of the total monomer repeats, respectively. The four dinucleotides are all composed entirely of AT/TA.

### Collinearity analysis of mitochondrial genomes in Fabaceae

**Fig 4:**
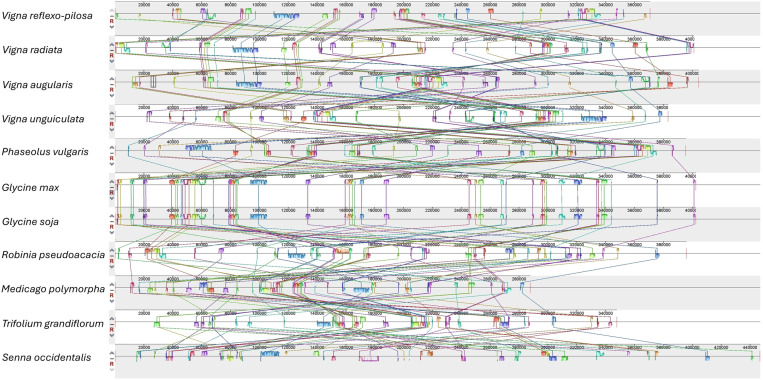
Collinearity analysis of legume mitochondrial genomes. Locally collinear blocks identified among four *Vigna* species and seven other plant species in Fabaceae. Each colored region represents a local collinear block revealing the similarity between genomes. The connecting lines represent as connected blocks to trace each orthologous region.

**Fig 5:**
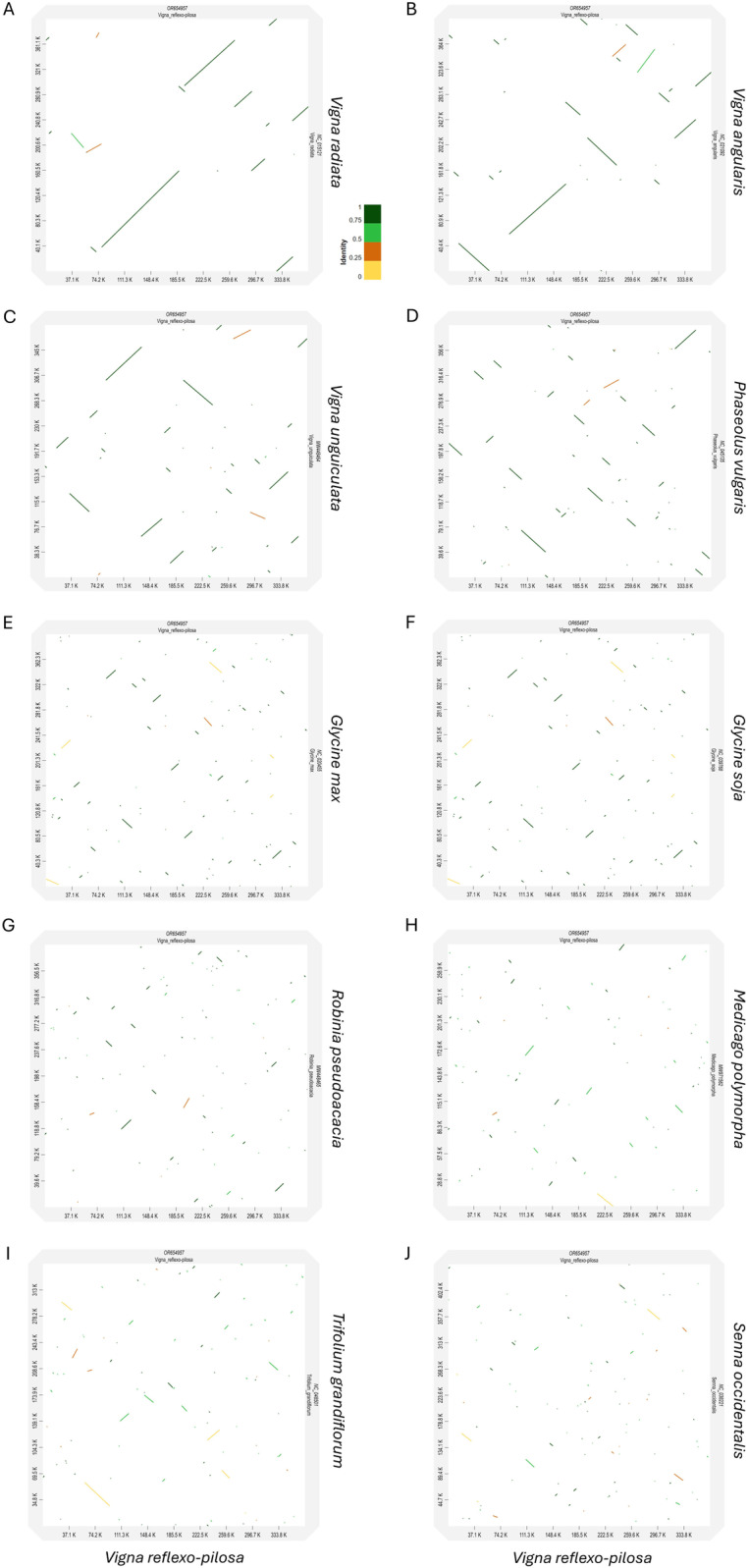
Dot plot analysis. Dot plot of four *Vigna* species and seven other plant species in Fabaceae. Horizontal coordinates in each box represent the mitochondrial *Vigna reflexo-pilosa* sequence, whereas vertical coordinates represent other mitochondrial sequences. Color lines denote the percentage of sequence identity.

Mitochondrial genome synteny among eleven plant species in the family Fabaceae, including four *Vigna* species, *P. vulgaris*, *G. max*, *G. soja*, *R. pseudoacacia*, *M. polymorpha, T. grandiflorum*, and *S. occidentalis*, was evaluated. We found numerous collinear blocks that contained long and short ortholog sequences (Fig 4). Most of them were rearranged among species, except *G. max* and *G. soja*. Notably, the mitochondrial genomes of *V. reflexo-pilosa* and *V. radiata* have undergone less genome rearrangements than other *Vigna* species. Furthermore, a dot-plot analysis was also performed (Fig 5). The results showed numerous collinear blocks between *V. reflexo-pilosa* and *V. radiata*, but others were short and fragmented.

Between *V. reflexo-pilosa* and other *Vigna* species, a total of six shared mitochondrial fragments (0.95–477.20 kb) were observed (S3 Table). Pi values of the six fragments ranged from 0.0088 (fragment 2) to 0.0159 (fragment 4). In addition, Pi values of the fourteen conserved genes in Fabaceae ranged from 0.0031 (*nad7*) to 0.0203 (*nad9*) (S4 Table). These results revealed low levels of divergence in the mitochondrial regions among *Vigna* species and plant species in Fabaceae.

### Comparative chloroplast genomes

**Fig 6:**
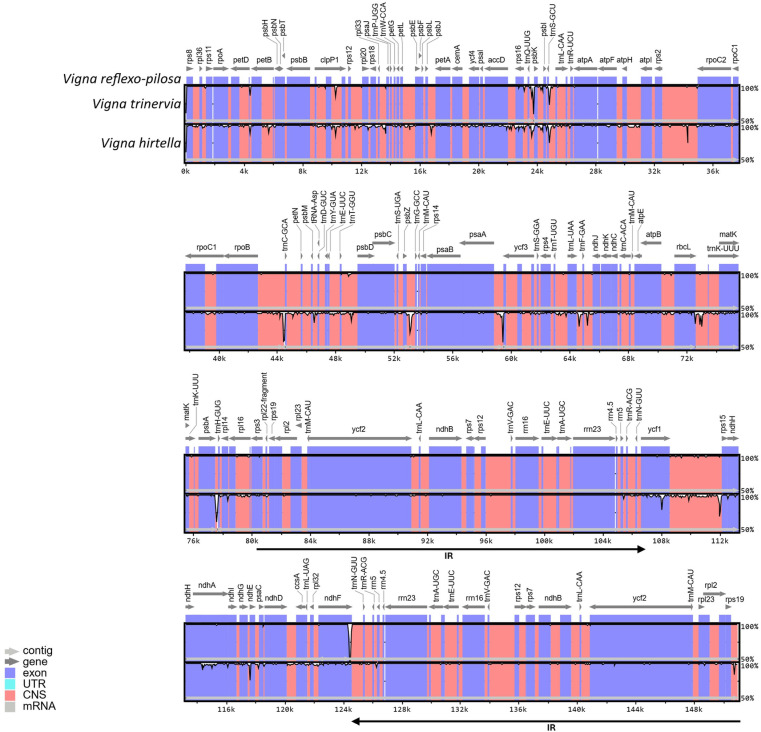
Alignment map of three *Vigna* chloroplast genomes in Fabaceae. Horizontal and vertical axes show genome position and a percentage sequence identity, respectively.

The differences among three *Vigna* chloroplast genomes were evaluated using mVISTA based on the annotated *V. reflexo-pilosa* chloroplast genome as a reference (Fig 6). The chloroplast genome of *V. reflexo-pilosa* was highly conserved with *V. trinervia* when compared with *V. hirtella*. The result showed a high divergence level between *V. reflexo-pilosa* and *V. hirtella* in several non-coding regions of LSC and SSC regions such as *rpoB*-*trnC*, *psbZ*-*trnG*, *psaA*-*ycf3*, *psbA*-*trnH*, and *ycf1*-*rps15*. Among the chloroplast genomes, a low divergence level was found in IR regions.

### Phylogenetic relationship of *V. reflexo-pilosa*

**Fig 7:**
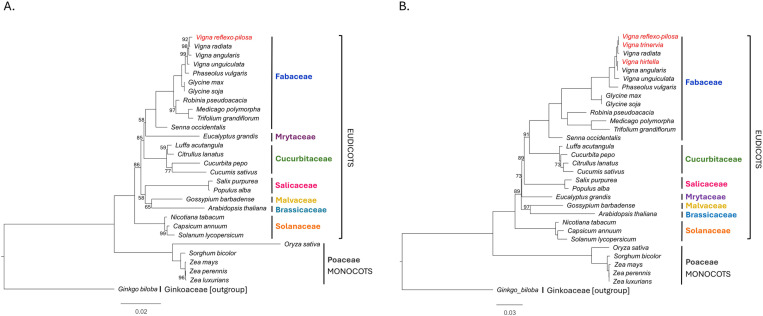
Evolutionary relationships of *Vigna reflexo-pilosa* and related species. (A) Maximum likelihood tree inferred from the concatenated data sets of 14 mitochondrial genes in 29 accessions (B) Maximum likelihood tree inferred from the concatenated data sets of 54 plastid genes in 33 accessions. Bootstrap support values less than 100% are denoted above branches.

To reveal the evolution of *V. reflexo-pilosa*, ML phylogenetic trees were performed using mitochondrial and chloroplast genes (Fig 7). Based on 14 conserved genes among 29 mitochondrial genomes, the ML tree showed that *V. reflexo-pilosa* was in the clade of Fabaceae and was closer to *V. radiata* than to *V. angularis* and *V. unguiculata* with high bootstrap values (>90%) as shown in Fig 7A. Based on 54 conserved genes among 31 chloroplast genomes (the 29 species with the analysis of mitochondrial genomes and two *Vigna* species: *V. trinervia and V. hirtella*), the ML tree revealed that *V. reflexo-pilosa* was also in the clade Fabaceae and was closer to *V. trinervia* than to *V. radiata*, *V. hirtella, V. angularis* and *V. unguiculata* with 100 bootstrap values (Fig 7B).

## Discussion

The mitochondrial genome of *V. reflexo-pilosa* was constructed using PacBio long-read data, resulting in a genome size of 370,913 bp with a GC content of 45.20%. The size of *V. reflexo-pilosa* mitochondrial genome was slightly smaller than the mitochondrial genome size of closely related species such as *V. angularis* (404,466 bp), *V. radiata* (401,262 bp), and *V. unguiculata* (383,314 bp) [8,12]. However, the overall GC and gene contents of the mitochondrial genomes in *Vigna* species are similar. The mitochondrial genome of *V. reflexo-pilosa* contains 31 unique protein-coding genes, which are consistent with other *Vigna* species such as *V. angularis* and *V. radiata* [8,12]. Additionally, the presence of two copies of the *atp9* gene was observed in the *V. reflexo-pilosa* mitochondrial genome, similar to that found in *V. radiata, V. angularis* and *Castanea mollissima* [8,12,44]. Our results revealed the absence of seven mitochondrial genes (*cox2, rpl2, rpl10, rps2, rps11, rps13,* and *sdh3*) in *V. reflexo-pilosa*, which are concordant with *V. angularis* and *V. radiata* [8,12]. Notably, *rpl2* is commonly lost in legumes, and *cox2* is lost in the subfamily Phaseolinae [10].

The chloroplast genome of *V. reflexo-pilosa* (150,967 bp), *V. trinervia* (151,226 bp), and *V. hirtella* (151,915 bp) were slightly different with the result of a previous study (153,169 bp, 151,161 bp and 151,564 bp, respectively) [18]. Their chloroplast genome sizes are similar to *V. radiata,* which was found to be 151,271 bp [15], and *P. vulgaris* was 150,285 bp [19]. The number of genes of the three *Vigna* chloroplast genomes are similar to the number of genes in other legume chloroplast genomes that contain 126 − 130 genes including 108 − 111 unique genes and 17 − 19 genes which are duplicated in the IR region. [13–17,19].

While mitochondrial genomes in most plants often contain shared genes with chloroplast genomes [14], our finding demonstrated there was no gene transfer from chloroplast to mitochondria of *V. reflexo-pilosa*. Both mitochondrial and chloroplast genomes encode a set of genes that allow them to synthesize their own proteins. The independence of the two genomes indicates that each has preserved the necessary capacities to perform its essential functions.

In angiosperms, RNA editing is a post-transcriptional process that modifies genetic information by converting C to U, which was found in mitochondrial transcripts [45–47]. Interestingly, RNA-editing events were found in 30 genes of the mitochondrial genome of *V. reflexo-pilosa*, which were also observed in other plants such as *Arabidopsis thaliana* [48] and *Calophyllum soulattri* [49]. RNA-editing in several genes such as *ccmFc, mttB, nad1, nad4L, nad5,* and *rps10* were consistent with presumed RNA-editing events in *V. angularis* [12] and *P. vulgaris* [10]. Remarkably, *mttB, nad1, nad4L*, and *rps10* genes were edited on the second site of the start codon, and the *ccmFc* gene encoded C-terminal region of *ccmF* was edited on the first site of the stop codon, which was found in other plants such as *Phaseolus vulgaris* [10], *Calophyllum soulattri* [49], and *Garcinia mangostana* [50]. The *ccmF* genes are essential components of the electron transport chain in mitochondria [51]; therefore, the RNA-editing event affects their functions. Indeed, RNA editing can lead to the start and stop codon generation and affect the structure of protein in plant mitochondria [52]. For example, the nucleotide in the early position of exon2 of *ccmFc* gene in wheat mitochondria changed from an asparagine codon to a stop codon, which may lead to change protein structure and function [51].

Comparison of three *Vigna* chloroplast genomes using mVISTA supported that *V. trinervia* is a maternal parent of *V. reflexo-pilosa* due to the high similarity of their chloroplast sequences, which is concordant with several previous studies based on gene and genome sequences [4,5,18].

Phylogenetic analysis of *V. reflexo-pilosa* based on mitochondrial and chloroplast protein-coding genes provides insights on its evolution. Our tree and previous trees in Fabaceae using mitochondrial or chloroplast genes had highly similar topologies [2,18]. For example, the phylogenetic relationships based on single copy chloroplast genes among 23 *Vigna* accessions, *V. reflexo-pilosa* and *V. trinervia* showed a monophyletic group, and *V. hirtella* showed a polyphyletic group with *V. reflexo-pilosa* and *V. trinervia* [18]. To contrast, phylogenetic trees based on nuclear genes or nuclear rDNA-ITS regions, *V. reflexo-pilosa* and *V. hirtella* showed a monophyletic group, whereas *V. trinervia* showed a paraphyletic or polyphyletic group with *V. reflexo-pilosa* and *V. hirtella* [2,5]. Therefore, *V. reflexo-pilosa*, *V. trinervia* and *V. hirtella* are high relationships based on the results of phylogenetic trees, confirming *V. reflexo-pilosa* and its parents. Therefore, phylogenetic trees could represent and reflect the evolutionary history of *V. reflexo-pilosa* and related species using the difference of markers.

## Conclusions

In this study, the complete mitochondrial genome of *V. reflexo-pilosa* and the chloroplast genomes of *V. reflexo-pilosa*. *V. trinervia* (female parent), and *V. hirtella* (male parent) were presented. The gene contents of the mitochondrial genome of *V. reflexo-pilosa* were similar to those of other *Vigna* species. There are 520 RNA-editing sites in 30 genes of the *V. reflexo-pilosa* mitochondrial genome. Comparison of mitochondrial genomes across Fabaceae species showed that the mitochondrial structure of *V. reflexo-pilosa* and *V. radiata* were similar than other Fabaceae species. In addition, high chloroplast sequence similarities were observed in *V. reflexo-pilosa* and *V. trinervia*, revealing maternal lineage. The phylogenetic analysis based on both mitochondrial and chloroplast genes showed that *V. radiata* is closely related to *V. reflexo-pilosa*. Thus, the complete mitochondrial and chloroplast genomes of *V. reflexo-pilosa* as well as its parents are a good source of genetic information for studying evolution and phylogeny in plants of the family Fabaceae.

## Supporting information

S1 Figure The complete chloroplast genome of *V. trinervia* and *V. hirtella.* Circular structure of (A) *V. trinervia* and (B) *V. hirtella* chloroplast genome. Known protein-coding genes, tRNAs and rRNAs are shown on the outside and inside of the circle. Colored genes were based on their functional groups.
(DOCX)

S1 Table List of repeats in the *V. reflexo-pilosa* mitochondrial genome. (XLSX)

S2 Table List of SSRs in the *V. reflexo-pilosa* mitochondrial genome. (XLSX)

S3 Table Nucleotide diversity of six shared mitochondrial fragments between *V. reflexo−pilosa* and three other *Vigna* species. (DOCX)

S4 Table Nucleotide diversity of 14 shared mitochondrial genes in *V. reflexo-pilosa* and ten reported plant species in the family Fabaceae. (DOCX)
