# Supplementary material for: Assembly and analysis of the complete mitochondrial and chloroplast genomes of Vigna reflexo-pilosa
Source: PLoS One. 2025 Jun 11;20(6):e0325243. doi: 10.1371/journal.pone.0325243 (PMC12157084; doi:10.1371/journal.pone.0325243)
Supplement: S1 Figure — Circular structure of (A) V. trinervia and (B) V. hirtella chloroplast genome. Known protein-coding genes, tRNAs and rRNAs are shown on the outside and inside of the circle. Colored genes were based on their functional groups. (DOCX) [file pone.0325243.s001.docx]

**
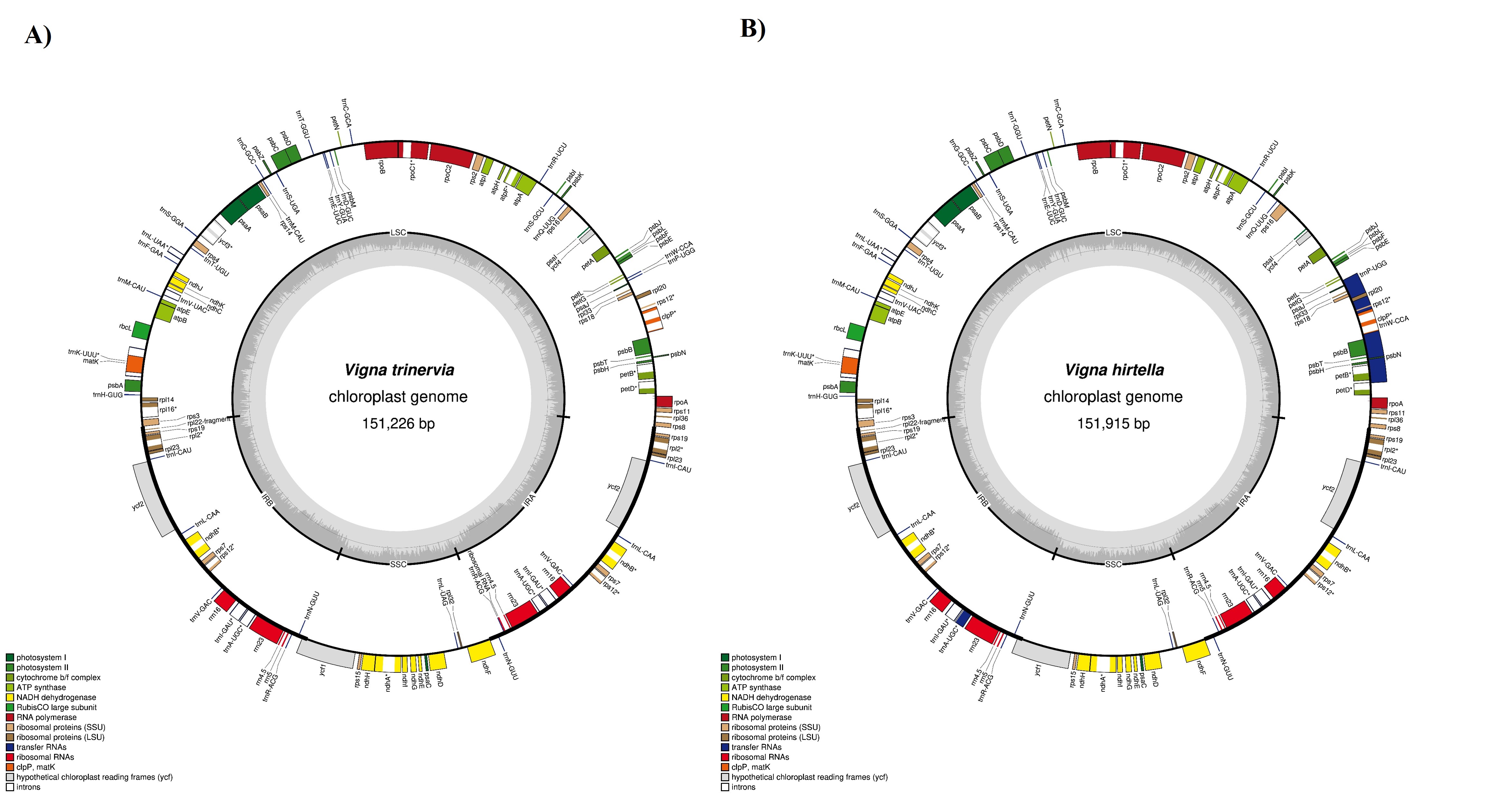
**

**S1 Figure.** **The complete chloroplast genome of *V. trinervia* and *V. hirtella.*** Circular structure of (A) *V. trinervia* and (B) *V. hirtella* chloroplast genome*.* Known protein-coding genes, tRNA genes, and rRNA genes are shown on the line.
