# Supplementary material for: Assembly and analysis of the complete mitochondrial and chloroplast genomes of Vigna reflexo-pilosa
Source: PLoS One. 2025 Jun 11;20(6):e0325243. doi: 10.1371/journal.pone.0325243 (PMC12157084; doi:10.1371/journal.pone.0325243)
Supplement: S3 Table — (DOCX) [file pone.0325243.s004.docx]

**S3 Table.** **Nucleotide diversity of six shared mitochondrial fragments between *V. reflexo-pilosa* and three other *Vigna* species.**

| **Fragment** | **Mitochondrial genome position** | **Number of sites** | **Nucleotide diversity (Pi)** |
| --- | --- | --- | --- |
| Fragment1 | *V. reflexo-pilosa*: 16,515−24,212  *V. radiata*: 178,302−189,155  *V. angularis*: 23,885−44,170  *V. unguiculata*: 196,655−212,730 | 139 | 0.00970 |
| Fragment2 | *V. reflexo-pilosa*: 36,112−50,245  *V. radiata*: 205,021−219,119  *V. angularis*: 822−23,161  *V. unguiculata*: 99,954−127,113 | 236 | 0.00886 |
| Fragment3 | *V. reflexo-pilosa*: 86,509−136,455  *V. radiata*: 58,600−107,110  *V. angularis*: 62,000−109,570  *V. unguiculata*: 299,619−349,563 | 611 | 0.00675 |
| Fragment4 | *V. reflexo-pilosa*: 279,312−280,296  *V. radiata*: 261,601−285,647  *V. angularis*: 318,610−332,190  *V. unguiculata*: 364,196−375,674 | 29 | 0.01591 |
| Fragment5 | *V. reflexo-pilosa*: 319,611−326,124  *V. radiata*: 390,769−401,262  *V. angularis*: 213,319−242,555  *V. unguiculata*: 134,485−160,153 | 103 | 0.00840 |
| Fragment6 | *V. reflexo-pilosa*: 358,262−370,913  *V. radiata*: 239,519−261,600  *V. angularis*: 296,502−318,609  *V. unguiculata*: 349,864−362,497 | 194 | 0.00813 |
