# Supplementary material for: Assembly and analysis of the complete mitochondrial and chloroplast genomes of Vigna reflexo-pilosa
Source: PLoS One. 2025 Jun 11;20(6):e0325243. doi: 10.1371/journal.pone.0325243 (PMC12157084; doi:10.1371/journal.pone.0325243)
Supplement: S4 Table — (DOCX) [file pone.0325243.s005.docx]

**S4 Table.** **Nucleotide diversity of 14 shared mitochondrial genes in *V. reflexo-pilosa* and ten reported plant species in the family Fabaceae.**

| **Gene** | **Number of sites** | **Nucleotide diversity (Pi)** |
| --- | --- | --- |
| *atp9* | 2 | 0.00444 |
| *ccmB* | 24 | 0.01366 |
| *ccmC* | 14 | 0.00680 |
| *cob* | 27 | 0.00788 |
| *cox1* | 25 | 0.00617 |
| *cox3* | 15 | 0.00732 |
| *nad3* | 4 | 0.00317 |
| *nad4* | 11 | 0.00208 |
| *nad6* | 21 | 0.01214 |
| *nad7* | 10 | 0.00313 |
| *nad9* | 32 | 0.02032 |
| *rps12* | 12 | 0.00989 |
| *rps3* | 52 | 0.01602 |
| *rps4* | 40 | 0.01554 |
